# The Decision Between Tooth Retention or Replacement with Implants: A Continuing Dilemma

**DOI:** 10.3390/dj13030099

**Published:** 2025-02-26

**Authors:** Eleni Bentour, Eirini Papamanoli, Ioannis K. Karoussis

**Affiliations:** National and Kapodistrian University of Athens, Thivon 2, 11527 Athens, Greece; ebentour@med.uoa.gr (E.B.); irenepap@med.uoa.gr (E.P.)

**Keywords:** periimplantitis, tooth prognosis, implantology, doubtful teeth, tooth retention

## Abstract

The global adoption of dental implants has significantly reshaped modern dental practices, with the market projected to reach USD 16 billion by 2029. However, despite high success rates, dental implants can still be prone to complications, particularly when underlying causes of tooth loss, such as periodontal disease and bone loss, are not addressed. This paper explores the biological and mechanical considerations in the decision-making process between preserving a tooth through periodontal therapy or opting for extraction and implant placement. It also highlights the importance of a holistic approach that includes assessing the patient’s oral health, periodontal status, and the biomechanical factors influencing tooth retention. Periodontal therapy has been proven to be highly effective, with both non-surgical and surgical therapies showing long-term efficacy in preserving natural teeth, especially in the presence of furcation involvement. Studies show that proper periodontal management, including regular maintenance therapy after the active therapy, significantly enhances tooth survival, even in cases of severe periodontitis. In contrast, dental implants, while effective, are not free of complications, mainly inflammatory peri-implant diseases, but also mechanical complications, which can compromise long-term outcomes. The paper reviews clinical studies on implant survival, demonstrating that periodontal therapy can sometimes offer a more cost-effective and biologically sound alternative to implant therapy, especially for teeth with severe attachment loss or furcation involvement. In conclusion, treatment decisions should be based on a comprehensive evaluation of clinical, biological, and patient-specific factors. By integrating regenerative therapies even in more compromised teeth and addressing the root causes of tooth loss, implant rehabilitation can be postponed for many years and offer a cost-effective and successful long-term treatment plan. This approach underscores the importance of individualized care in the evolving landscape of restorative dentistry and implantology.

## 1. Introduction

Implants as a treatment option have been widely adopted in dental practices on a global level. This global adoption underscores the relevance and importance of this field in contemporary dental care. In 2023, it was estimated that around 12–15 million dental implants were placed annually, a testament to the widespread acceptance of this treatment modality. By 2029, the global market for dental implants and prosthetics is projected to reach USD 16 billion, with a growth rate of approximately 7.5% per year [1]. Advanced implant designs have been created to address various challenging clinical situations. While dental implants have high survival rates, a substantial number of complications also follow their placement.

Frequently, the fundamental causes of tooth loss—such as periodontal disease, bone loss, or other underlying biological issues—are overlooked in the process of tooth replacement. Instead, a more mechanical approach is frequently chosen, focusing on the technical aspects of implant placement, e.g., bone sufficiency, without addressing the biological or biomechanical factors contributing to the original tooth loss.

A simplified approach prioritizes the straightforward replacement of a missing tooth with an implant, sometimes neglecting the need to understand and rectify the conditions that led to tooth loss. This might involve ensuring the implant is placed favorably and easily but does not necessarily consider how to prevent future issues or if the tooth was to be replaced in the first place.

Conversely, a biological perspective would involve a comprehensive evaluation of the patient’s oral health, including the condition of the periodontal tissues and how these factors impact the success of the implant if one opts for extraction. This approach aims to address the root causes of tooth loss to not only ensure the immediate success of the implant but also to promote long-term oral health and function.

By integrating a thorough understanding of the patient’s oral biology and biomechanics, dental professionals can better tailor treatments to individual needs, maintaining as many teeth as possible and improving both the outcomes of implant procedures, if deemed necessary, and overall patient well-being.

With advancements in restorative dentistry and implantology, the selection between tooth preservation or extraction and implant placement has become increasingly complex. As treatment options evolve in both fields, understanding the criteria that influence prognosis and treatment outcomes is essential for ensuring optimal care. This paper aims to explore the key considerations in choosing between attempting to retain a tooth or proceeding with extraction and implant placement, focusing on clinical, biological, and patient-centered factors.

## 2. Teeth Response to Periodontal Treatment

The primary objective of periodontal therapy is to preserve the health, function, and comfort of as many teeth as possible. Different tooth types respond differently to treatment [2].

The effectiveness and long-term benefits of periodontal treatment are generally accepted. Findings from the study by Kaldahl et al. (1996) demonstrate that different therapeutic modalities—ranging from non-surgical procedures such as scaling and root planning to more invasive surgical techniques like pocket elimination surgery and modified Widman flap surgery—can significantly reduce probing depths and improve clinical attachment levels in patients with periodontitis, as well as secure long-term survival of those teeth [3]. This improvement highlights the adaptability of periodontal therapy to various disease severities. The long-term evaluation of these therapeutic modalities confirms that periodontal treatment, particularly when combined with regular maintenance care, is highly effective in preserving natural teeth over extended periods. Even in more advanced cases requiring surgical intervention, the retention of teeth remains high. In addition, respective surgical treatments generally offer more substantial and sustained improvements in periodontal health, especially in deep periodontal pockets, compared to non-surgical therapy or access-flap surgery. However, even non-surgical treatments can be effective for managing less severe cases of periodontal disease [3]. Longitudinal studies have revealed the efficiency of both non-surgical and surgical periodontal therapy. Tooth loss, which was usually the primary outcome of long-term retrospective evaluations, is impressively low since McFall, who followed periodontal patients for 15–29 years in a periodontal practice, reported that out of 832 teeth receiving surgical therapy, 15.7% were lost. Moreover, in a total of 2627 teeth, 9.9% were, lost and out of 1795 teeth treated non-surgically, 7,1% were lost [4]. This, of course, does not imply that non-surgical therapy is more effective in tooth retention, but that the application of the appropriate treatment steps during the active phase is crucial and effective in the long-term maintenance of the dentition. Interestingly, 80% of the teeth lost belonged to 23 out of 100 patients, which implies the role of disease activity, systemic factor, and also compliance. If one were to directly compare non-surgical and surgical methods, the results of the classical literature reveal that both are effective in disease control, as shown by Philstrom et al. (1981), who compared the effects of subgingival instrumentation to subgingival instrumentation with a modified Widman flap procedure in a split-mouth design. The different sides were then compared regarding the clinical indices change [5]. The results indicated that no significant differences in plaque or inflammation occurred between the two procedures irrespective of initial pocket depth. However, other studies reveal differences according to the initial pocket depth. Kaldahl et al. have performed a prospective study comparing the results of supragingival debridement, subgingival instrumentation, modified Widman flap, and apically repositioned flap with osseous surgery [3]. For pocket depths between 4 and 6 mm, subgingival instrumentation and Widman flaps were equally effective in clinical index improvement; however, for deep pockets > 7 mm, only osseous surgery resulted in a significant improvement in clinical parameters, which was sustained throughout the follow-up period. However, at this point, it must be highlighted that in the group of osseous surgery, teeth or roots that were assigned as having a poor prognosis after the osteoctomy/osteoplasty were immediately extracted. This perhaps is the reason why earlier studies exhibited similar results for moderate and deep pockets with both scaling and root planning and Widman flap surgery. The most important thing, however, is the level of oral hygiene, which is more critical for resulting probing depth and attachment levels than was the mode of therapy [6]. Sites that, during the maintenance period, were free of plaque were associated with shallow pockets and maintained attachment levels. The addition of systemically administered antibiotics, especially amoxicillin and metronidazole, adds a further benefit in deep and moderate pocket reduction and may also reduce the need for additional therapy, according to earlier and recent systematic reviews. Despite the added benefit, the risk of overprescription and development of antibiotic resistance, which is a serious public health issue, is the reason that the new EFP guidelines have an open consideration when it comes to antibiotics and the discrimination of periodontitis grades, suggesting their use in cases with grade c periodontitis in young individuals, especially in the incisor-molar pattern [4,5].

According to Ramfjord and colleagues, tooth groups have an impact on how different treatments work when the relationship between initial probing depth and tooth type is taken into account. Maxillary molars exhibited a greater long-term increase in pocket depth and a less significant short-term decrease in pocket depth, which may be partly related to furcation involvement [2]. Furcation involvements are a highly common occurrence in cases of periodontitis, with a reported prevalence of approximately 30–50% in patients with the disease [7].

Many studies have been conducted to determine the response of teeth with furcation involvement to periodontal treatment, both non-surgical and surgical. In 1978, Hirschfeld and Wasserman conducted a landmark long-term study examining tooth loss in 600 patients who had undergone periodontal treatment. The study aimed to assess the effectiveness of periodontal therapy in maintaining teeth over an extended period, with the average duration of periodontal maintenance therapy being 22 years. It must be noted that while the majority of the patients (76.5%) were initially classified as having advanced periodontitis (probing pocket depth > 7 mm and at least one furcation involvement), their correspondence to the supportive periodontal therapy (SPT) program played a catalytic role in the preservation of their natural dentition. Over 22 years, only 7.1% of all teeth were lost due to periodontal reasons. Of the 1464 teeth with furcation involvements from the beginning, 460 were lost (31.4%), with 240 of those coming from one-sixth of the patients who deteriorated most. More specifically, of the 867 maxillary teeth diagnosed with furcation involvement, 284 were lost. In comparison, out of 597 mandibular teeth with the same condition, 176 were lost. In other words, the survival rate of teeth with furcation involvement over a mean period of 22 years was 68.6%.

Half of the sample, or 300 out of 499 patients in the group that responded well in the post-treatment course (well-maintained group), did not experience any tooth loss during the average 22-year period. Only 31.1% of the teeth that were initially marked as questionable because of furcation involvement, severe mobility, or deep pocket depths were eventually lost [7].

A 2016 systematic review from Nibali et al. evaluated the survival of molars with furcation involvement by examining 21 studies [8]. According to the review, the survival rates of teeth following surgical treatment varied depending on the type of study: 94% to 100% after 4 to 7.5 years for regeneration studies, 89% after 5.8 years for tunneling studies, 79% after at least 4 years for root resection studies, and 43% to 100% over 5 to 53 years for studies using combined therapies. The average annual tooth loss in molars without furcation involvement in these studies was 0.01; for those with furcation involvement, it was 0.02. In the follow-up period, 8%, 18%, and 30% of teeth with furcation degree I, II, and III, respectively, were lost [8]. The relative risk of tooth loss during maintenance therapy attributable to Furcation Involvement [FI] was 1.46 (95% Confidence Interval [CI] = 0.99–2.15, *p* = 0.06) for studies up to 10 years and 2.21 (95% CI = 1.79–2.74, *p* < 0.0001) for studies with a follow-up of 10–15 years. A gradual increase in the risk of tooth loss was observed for molars with degree II and III FI.

Recent systematic reviews on furcations have concentrated on short-term results, finding that degree II furcations can be successfully treated with reconstructive surgery, particularly when combined with regenerative therapy [9], and that they significantly improve six months after access flap surgery [10]. A survival rate of over 90% within 5–9 years has been recorded for molars with furcation involvement treated without surgery, with outcomes varying based on treatment methods and levels of disease severity [11]. Conservative non-surgical therapy for multirooted teeth with furcation involvement is generally effective in preventing the progression of disease in degree I lesions. However, as the disease advances and more attachment loss occurs, this treatment faces challenges, particularly in fully removing calculus [12].

More specific information on the survival of multirooted teeth with furcation involvement with different treatments can be found on Huynh Ba et al.’s systematic review. The review included twenty-two publications. Based on ten studies, after surgical procedures, such as gingivectomy, apically repositioned flap with or without osseous recontouring, etc., the survival rate of teeth with furcation lesions was 43.1–96% after an observation period of 8 to 22 years. In Dannewitz et al.’s retrospective study, at approximately nine years, the survival rate was 93.6% after non-surgical and surgical therapy [13].

Huynh-Ba et al.’s systematic review reported survival rates for furcation-involved teeth after tunneling procedures and surgical resective therapy (including root resection and/or root separation), which ranged from 42.9% to 92.2% over a 5- to 8-year observation period and from 62% to 100% over a 5- to 12-year period, respectively. The most common complications following resective surgery were recorded to be vertical root fractures and endodontic complications [9].

K. Jepsen et al. introduced a novel treatment protocol for severely furcation-involved maxillary molars, focusing on vital root resection as an alternative to conventional root canal therapy and extraction. Their randomized controlled trial included 15 maxillary molars presenting double/triple class II or single/double class III furcation defects. These molars also exhibited advanced vertical bone loss around one root.

The procedure involved performing a deep pulpotomy using calcium silicate-based cement, followed by the resection of the affected roots after 4 weeks. The study emphasized the importance of preserving tooth vitality during the treatment, as all treated teeth remained sensitive to pulp testing throughout the follow-up period of 3 to 7 years.

Vital clinical outcomes included a reduction in pocket probing depths to ≤5 mm across all treated teeth; significant improvement in furcation status; no signs of increasing tooth mobility, periapical pathology, or adverse clinical and radiographic findings over the extended observation period; patients expressed satisfaction with the therapy, reporting positive patient-reported outcomes [14].

This protocol demonstrated long-term success for up to 7 years, offering an effective alternative for maintaining maxillary molars affected by severe furcation involvement while reducing costs and complications associated with endodontic therapy intraoperatively or later.

Regeneration surgery has demonstrated significant benefits for treating teeth with poor prognosis, even those with severe attachment loss. A 10-year randomized controlled trial by Cortellini et al. (2020) compared periodontal regeneration (PR) with tooth extraction and replacement with titanium implants (TER) in patients with attachment loss to or beyond the apex of the root [15]. The study included 50 patients with stage III or IV periodontitis, randomized to receive either PR or TER with implants or tooth-supported fixed partial dentures. For the regeneration procedures, the authors state to have used different kinds of resorbable or non-resorbable membranes, emdogain, and particulate grafts per case. The results revealed that both treatment modalities had high survival rates, with 88% in the PR group and 100% in the TER group. Complication-free survival was comparable between the groups, ranging from 6.7 to 9.1 years for PR and 7.3 to 9.1 years for TER, with no significant difference. Notably, the PR group achieved a 10-year attachment gain of 7.3 ± 2.3 mm and maintained residual probing depths of 3.4 ± 0.8 mm. Additionally, PR was associated with significantly lower long-term costs compared to TER. Patient responses regarding their outcomes and quality of life related to oral health both showed enhancement in both groups, with PR demonstrating a beneficial cost-effectiveness balance.

The study concludes that periodontal regeneration can notably alter the prognosis of severely compromised teeth and presents a cost-effective alternative to tooth extraction and replacement. While the complexity of the procedure limits its application to the most challenging cases, it provides compelling evidence of the efficacy and advantages of PR for deep intra-bony defects [15].

Nibali and colleagues conducted a systematic review to compare regenerative therapy and access flap surgery defects in terms of clinical, radiographic, and patient-reported outcomes for treating intra-bony defects [16]. A total of 79 randomized clinical trials were selected and analyzed. These studies, published between 1990 and 2019, involved 3042 patients and 3612 intra-bony defects. The comparisons comprised GTR with resorbable and non-resorbable membranes (GTR-R, GTR-NR), biomaterials such as emdogain (EMD), platelet-rich factor (PRF), demineralized freeze-dried bone allograft (DFDBA), and deproteinized bovine bone mineral (DBBM). The findings showed that regenerative techniques, especially those involving enamel matrix derivatives (EMD) and guided tissue regeneration (GTR), consistently result in better outcomes in terms of clinical attachment level gain and defect fill compared to access flap surgery alone. When it comes to the comparison between the different regenerative techniques, GTR-R and GTR-NR have no significant differences in terms of attachment and bone gain, and the combination of EMD with bone grafts is superior to the addition of EMD alone in terms of attachment and bone gain. Finally, the addition of PRP (platelet-rich plasma) to DFDBA significantly improves clinical attachment and bone gain, but not pocket reduction.

Slowly, a growing number of evidence supports the use of alternative biological agents for periodontal regeneration, predominantly hyaluronic acid. Hyaluronic acid (HA) is a naturally occurring non-sulfated glycosaminoglycan with a high molecular weight of 4000–20,000,000 Da and is a key element in the gingiva, connective tissue, and periodontal ligament, and in the hard tissue, such as alveolar bone and cementum. It can play a regulatory role in inflammatory response: the high-molecular-weight HA synthesized by hyaluronan synthase enzymes in the periodontal tissues undergoes degradation to lower molecular weight molecules in chronically inflamed tissue, such as gingival tissue inflammation or in the postoperative period after surgery. The reason why high-molecular-weight HA is preferred is because it suppresses pronounced immune reactions such as exacerbated inflammation. A preclinical study in dogs compared open flap debridement (OFD) with OFD + cross-linked HA, OFD + collagen matrix (CM), and their combination in two-wall surgically created defects. The HA (2.43 ± 1.25 mm) and HA/CM (2.60 ± 0.99 mm) groups yielded statistically significant (P < 0.05) greater formation of new attachment (i.e., linear length of new cementum adjacent to newly formed bone, with inserting collagen fibers) compared with the OFD (0.55 ± 0.99 mm) group. Among the four treatment groups, the HA/CM group demonstrated the highest amount of regenerated tissues, although no statistically significant differences in any of the histometric parameters were observed between the HA and HA/CM groups. A recently published systematic review with meta-analysis by Ostos-Aguilar explored the improvement in attachment levels, pocket depth reduction, and bone gain by the addition of HA in combination with bone grafts, compared to bone grafts alone in regenerative processes. The addition of HA to OFD resulted in greater improvement in attachment gain (mean difference [MD]:1.00; 95% confidence interval [CI]:0.65–1.35; n = 2) and pocket reduction (MD: 0.76; 95%CI: 0.34–1.17; n = 2) at 6 months, but its addition to bone grafts only revealed a difference in radiographic bone fill (MD: 0.57; 95%CI: 0.15–0.99; n = 2) at 12 months.

Periodontal regeneration may also benefit molars with furcation-related defects, as is revealed in Jepsen et al.’s systematic review and Bayesian network meta-analysis [17]. The study analyzed 20 randomized controlled trials involving 575 patients and 787 defects, consistently demonstrating the superiority of regenerative techniques over open flap debridement. Regenerative methods included GTR with resorbable or non-resorbable membranes (GTR-RES, GTR-NONRES), bone replacement grafts (BRG), emdogain (EMD), or their combination, achieving between 0% and 60% furcation closure rates and conversion to class I furcations in 29% to 100% of cases. Moreover, these techniques resulted in significant horizontal and vertical bone level gains (1.6 mm and 1.3 mm, respectively) and reduced probing pocket depths (1.3 mm). Bone replacement grafts had the highest probability (61%) of being the most effective for horizontal bone level gain. In contrast, non-resorbable membranes combined with bone replacement grafts (BRG) ranked best for vertical attachment gain and probing depth reduction. Furcation improvement (FImp) was obtained in 19 out of 30 (63%) surgically treated defects for BRG, 48 out of 58 (83%) for EMD, 12 out of 14 (86%) for BRG + EMD, 68 out of 114 (60%) for GTR-NONRES, 23 out of 45 (51%) for GTR-NONRES + BRG, 113 out of 173 (65%) for GTR-RES, 22 out of 37 (59%) for GTR-RES + BRG, 10 out of 10 (100%) for GTR-RES + BRG + EMD, and 3 out of 55 (6%) for OFD. This study underscores the clinical benefits of regenerative surgery for class II furcation defects, surpassing open flap debridement in long-term success and effectiveness [17].

Huynh Ba et al.’s systematic review analyzed four publications on the topic regarding the survival of multirooted teeth after guided tissue regeneration. The survival rate ranged from 83.3% to 100% for an observation period of 5 to 12 years [11].

For these rates to be more comprehensible, one must consider the severity of the furcation lesion on a horizontal and vertical level, which will undoubtedly also affect the decision between a regenerative or respective approach after all.

To emphasize the significance of SPT in the management of molars with degree III furcation involvement treated with tunneling procedures, the findings from the study by Nibali et al. offer valuable insights [18]. The study, conducted on 102 molars with degree III furcation involvement treated by tunneling, found that the overall tooth loss rate was 29.4% over an average follow-up period of nearly eight years. This means that most molars with class III furcation, mostly of subclass A, survive almost ten years post-surgery. The analysis revealed that irregular or infrequent SPT was significantly associated with increased tooth loss, with a notable difference observed between patients who received regular SPT and those who did not.

This finding highlights that while tunneling can be an effective treatment for degree III furcation involvement, the success of such interventions is highly contingent upon the maintenance of regular SPT. As to the reasons for tooth loss, evidence shows that it may be of periodontal etiology. However, caries also needs to be strongly considered when opting for a tunneling approach.

Two more studies highlight the impact of not undergoing SPT on tooth loss, especially in molars with furcation involvement.

Bjorn and Hjort (1982) followed 221 individuals over 13 years without specific periodontal treatment. They found that bone loss in furcation areas increased from 18% to 32%, with 9% of molars lost, though only 2.5% were due to progressive furcation lesions [19].

Nibali et al. (2017) studied 2333 individuals over 11 years and reported that molars with FI had significantly higher tooth loss rates [20]. Molars without FI had a 5.6% loss rate, while those with degree III FI saw a 55.6% loss rate. Periodontitis patients showed even more significant losses, with 47.3% of those with severe periodontitis losing at least one molar.

In their long-term evaluation of periodontal therapy, Kaldahl et al. investigated the incidence of periodontal sites experiencing breakdown after treatment [21]. While periodontal therapy is generally effective in maintaining tooth health over the long term, a subset of treated sites may still experience breakdown. Factors that may influence site breakdown include the severity of initial disease, oral hygiene, maintenance therapy, smoking, and systemic health. Consistent and long-term follow-up care to manage and potentially prevent disease recurrence is an important key to the maintenance of a successful treatment.

Regardless of the treatment modality, maintenance of periodontal health requires continued care, with regular periodontal maintenance visits playing a crucial role in preventing the recurrence of disease and further site breakdown. Patients who adhere to post-treatment care plans have a higher chance of retaining their natural teeth and maintaining their periodontal health for many years after the initial treatment.

These findings underscore regular SPT’s importance in preserving teeth, especially those with furcation involvement. Without regular periodontal care, molars with FI are more likely to be lost, demonstrating the critical role of continuous maintenance in managing periodontal disease and preventing tooth loss.

## 3. Implant Success and Survival

In recent years, numerous meta-analyses have demonstrated that implant therapy is both effective and reliable [22,23,24,25,26,27]. Five-year cumulative survival rates range between 90.5% and 100%, with a failure rate of 0 to 2 per 100 implants. Similarly, 10-year cumulative survival rates range from 85.5% to 100%, with an estimated failure rate of 0 to 1.56 per 100 implants [17,19]. However, implant survival is no longer considered the primary indicator of success. Instead, the absence of technical or biological complications and high levels of patient satisfaction are now seen as the preferred measures of success. Acknowledging biological and aesthetic complications in implant therapy is frequently reported and is essential.

Peri-implantitis is a plaque-related pathological condition that affects the tissues surrounding dental implants. It is defined by inflammation in the peri-implant connective tissue and progressive loss of supporting bone [28]. Peri-implantitis is not an uncommon condition. According to a systematic review from 2015 that includes 11 studies, the weighted mean prevalence of peri-implant mucositis and peri-implantitis was estimated at 43% (CI: 32–54%) and 22% (CI: 14–30%), respectively [29]. Another cross-sectional study assessed the prevalence of moderate to severe peri-implantitis to be 15% [30]. Alongside the increasing number of dental implants placed each year, peri-implantitis has become an escalating concern for public health. It has been suggested that the progression of peri-implantitis follows a non-linear and accelerating manner, with the onset occurring within the first three years of implant function in most cases [31]. In moderate and advanced cases of peri-implantitis, non-surgical therapy fails to fully resolve the condition, making surgical intervention necessary [32].

The basis of pathogenesis and substrate–bacteria interactions are concomitantly similar but also very different from that of periodontal diseases. Classical studies of Lindhe and Zitzmann have demonstrated in vivo the microbial nature of peri-implant inflammatory lesions, while Pontoriero also showed the initiation and reversibility of peri-implant mucositis, similarly to the experiments of Loe for gingivitis in clinical conditions. All therapeutic attempts utilized the knowledge available for the treatment of periodontitis, yet a growing number of scientific evidence reveal substantial differences in peri-implant disease characteristics. According to Kotsakis and Olmedo, the first parameter that distinguishes peri-implantitis from periodontitis is the rampant rate of progression of the first. The explanation could lie in the difference in the initial colonizers in peri-implant tissues, which is strongly associated with the different substrate the implant poses, compared to the tooth. An implant surface, following exposure to water or oxygen, is covered by a thin oxide layer that renders the material bioinert and is very different from the cementum surface characteristics. According to the available literature, *Streptococcus* spp. employ their surface milieu, including adhesins, to colonize implant surfaces almost immediately after exposure to the oral environment. While *Streptococcus* spp. also have major roles in adhesion to organic dental substrates, the microenvironment created by implant surfaces creates a very different ecological succession. The paper of Kotsakis also links the possible resistance to antibiotics in peri-implant pathology to these differences. The authors explain how the different substrates affect the colonizers that succeed *Streptococcus* spp., which generally translates to a different composition of the biofilms. Interestingly, the clinical studies of Renvert et al., Blanco and Linares, and Hakkers et al. on the effect of systemically administered antibiotics in the non-surgical therapy of peri-implantitis all concluded the lack of important clinical benefit of the addition and also the lack of elimination of the need for additional therapy. Titanium dissolution products also affect the microbiome, as demonstrated by Daubert et al. Specifically, titanium concentrations in plaque were inversely correlated with species richness. Furthermore, variations were found in the structure of the microbiome community, not only between disease and health but also when high titanium concentrations were present [33].

In 2007, Schwarz et al. proposed a classification system for peri-implant bone defects based on the bone loss patterns observed around 40 implants. In this system, a class I defect is characterized by a buccal dehiscence and/or the presence of an intra-bony defect, while a class II defect is defined by horizontal bone loss. Class I defects are further divided into subclasses (class Ia to Ie) depending on the presence or absence of supporting bone walls and/or intra-bony defects at the four implant aspects: buccal, mesial, oral, and distal [30]. According to this study, 55,3% of the intra-bony defects were reported as circumferential (class Ie) [34]. In 2019, Monje et al. published their findings from a cone-beam computed tomography study on 158 eligible peri-implantitis implants and proposed a new classification that included an additional third category: class I—intraosseous; class II—horizontal; class III—a combination of class I and II. These were then subclassified into (a) dehiscence, (b) 2/3-wall, and (c) circumferential-type defect [35]. In contrast to Schwarz’s study, Monje et al. found class Ic to be the most frequent defect morphology (55% prevalence). Specifically, based on an a priori power calculation, 332 implants were screened in 47 peri-implantitis patients. Of these, 158 peri-implantitis implants were eligible. The most prevalent defect morphology type was class Ib (55%), followed by class Ia (16.5%) and class IIIb (13.9%). On the contrary, the less frequent defect was class II (1.9%). The most frequent degree of severity was M (50.6%), with S (10.1%) being the least prevalent. Buccal bone loss was significantly greater compared to the other bony walls in class I and class III defects. Age was associated with the type of defect. Age and smoking habit were associated with the morphology of the defects, while smoking habit, type of prosthesis, and distance to adjacent implant were associated with the severity of the defects (vertical bone loss). The discrepancy between the two studies may be attributed either to the smaller patient cohorts (≤40 implants) in earlier research or the assessment method used in the later study, which may introduce bias, especially in cases with thin alveolar bone.

In this regard, the configuration of peri-implant bone defects plays a key role in determining the appropriate treatment approach, whether resective, regenerative, or a combination of both, and it greatly influences the success of the surgical procedure. In a 2014 systematic review of seven studies, Heitz-Mayfield and Mombelli examined the success of peri-implantitis treatment over a 12-month period. They concluded that while most patients experienced favorable short-term outcomes, non-resolution, disease progression, or recurrence remained possible [36]. A more recent systematic review by Roccuzzo et al. calculated the implant survival following peri-implantitis treatment to be 81.73–100% at 3 years for seven studies, 74.09–100% at 4 years for three studies, 76.03–100% at 5 years for four studies, and 69.63–98.72% at 7 years for two years [37]. Long-term follow-up periods are necessary to properly evaluate the stability and effectiveness of treatment outcomes throughout time [36]. Additionally, an increased incidence of peri-implantitis at the implant level was connected with less frequent supportive care. History of periodontal disease complicated this finding [30].

A plethora of studies have demonstrated that in the same patient, the microbiota around teeth and implants presents many similarities [38,39,40,41]. The microbiota present in the oral cavity prior to implant placement plays a crucial role in forming the composition of the peri-implant microflora. Moreover, peri-implantitis lesions tend to have a microbial profile that resembles the bacteria found in chronic periodontitis, with a dominance of Gram-negative anaerobic bacteria such as *Porphyromonas gingivalis*, *Tannerella forsythia*, and *Treponema denticola* [42].

There has been a proven connection between periodontal and peri-implant conditions, implying that “the rate of progression of attachment loss adjacent to teeth and implants is similar in a given patient” [43]. This lends credence to the theory that a higher risk of developing periodontitis may also raise the risk of developing peri-implantitis.

In a 10-year prospective study by Karoussis et al., key findings highlight the relationship between periodontal and peri-implant conditions, particularly in partially edentulous patients [44]. The study evaluated 179 implants, all placed after comprehensive periodontal treatment. The initial cohort of patients had a total of 1770 teeth. Over the 10-year observation period, 87 natural teeth were extracted, resulting in a survival rate of 95% for the remaining teeth. This equates to a mean tooth loss rate of approximately one tooth per patient, or 0.1 per year. Despite some loss, these findings underscore the high overall survival rate of natural teeth over the extended observation period.

The study by Karoussis et al. found a strong correlation between periodontal health at natural teeth sites and the health of peri-implant tissues over a period of approximately 10 years [44]. This means that clinical and radiographic parameters—such as probing depth, attachment loss, and bone levels—used to assess the health of periodontal structures at natural teeth sites were also predictive of similar parameters around dental implants. In essence, the condition of a patient’s natural teeth, particularly regarding gum and bone health, was closely associated with the long-term health of their dental implants. The study highlights that poor periodontal conditions can influence peri-implant conditions, demonstrating the importance of overall oral health in the success of implants over time.

Research indicates that while implant survival rates in both the short and long term do not show significant differences between individuals with a history of chronic periodontitis and those with healthy periodontal conditions, patients with chronic periodontitis tend to experience more severe long-term outcomes. Specifically, they are more likely to exhibit deeper probing pocket depths, greater marginal bone loss around the implants, and a higher incidence of peri-implantitis compared to periodontally healthy individuals [45]. At this point, evaluating the proportion of patients and implants that follow peri-implantitis recurrence after their enrollment in supportive peri-implant therapy (SPIT) is particularly important. For this reason, numerous studies have taken place.

In the study conducted by Costa et al. (2012), the impact of SPIT was examined over a 5-year period to assess its effectiveness in preventing the progression of peri-implant mucositis to peri-implantitis. The study followed 80 patients who were initially diagnosed with peri-implant mucositis. These individuals were divided into two groups: one receiving regular preventive maintenance (at least five dental visits during the study period) and the other not receiving preventive care [46].

In the group that did not receive preventive maintenance, the incidence of peri-implantitis was alarmingly high, reaching 43.9%. The group that adhered to regular maintenance visits still presented an 18.0% incidence rate of peri-implantitis [46]. While the incidence of disease progression was significantly lower in the group receiving preventive care, the 18% recurrence rate in this group remains clinically significant and should not be underestimated. This emphasizes that even with consistent, supportive care, patients remain at risk of peri-implantitis, and the preventive program, although effective in reducing risk, is not infallible. This emphasizes the need for rigorous, long-term follow-up and individualized treatment strategies to effectively manage patients at risk for peri-implant disease.

In addition, clinical parameters such as bleeding on probing and pocket probing depths were notably worse in the group without maintenance. Furthermore, the study noted significant differences in peri-implant bone loss between the two groups. Interestingly, implant survival rates were lower in the non-maintenance group (98.4%) compared to the group with preventive care (99.37%), indicating preventive maintenance’s protective role in overall implant longevity only within the first 5 years.

Heitz-Mayfield et al. studied the outcomes of supportive peri-implant therapy from twenty-four patients with 36 dental implants diagnosed with peri-implantitis [47]. A successful treatment outcome was considered with the following criteria: implant survival, absence of peri-implant probing depths ≥ 5 mm with concomitant bleeding/suppuration, and absence of progression of peri-implant bone loss. At the 5-year evaluation, 15 out of 24 patients, or 63% of the total population, had successful treatment outcomes. In other words, 37% of the patients had experienced disease recurrence. Moreover, in only 42% of implants (15 out of 36 implants), peri-implantitis had fully resolved [47]. In this context, Charalampakis et al. (2011) found that disease recurrence or progression occurred in about 55% of patients over 9 months to 13 years. Smoking and smoking intensity were linked to treatment failure [48].

Disease recurrence may still occur regardless of the consistency of the SPIT program, potentially leading to implant failure or the need for removal [49]. Monje and Nart, in 2024, conducted another retrospective study [50]. The study examined data from 132 patients with 896 implants, all monitored for at least 12 months after peri-implantitis treatment. Twenty-eight patients identified as smokers. Among the implants studied, 99 were removed due to an unfavorable prognosis, while 397 received treatment for peri-implantitis. Disease recurrence occurred in 28 patients, which translates to 20% at the patient level and 10% at the implant level. Of the implants that experienced recurrence, 57.5% showed progressive bone loss, while 42.5% had deep pockets with significant bleeding on probing. Factors such as smoking, a baseline diagnosis of grade C periodontitis, and being male were significantly associated with a higher likelihood of recurrence [50].

In a 3-year prospective cohort study, Mercado et al. showed that after surgical reconstructive treatment for peri-implantitis, around 44% of the cases were unsuccessful, exhibiting progressive bone loss, residual pockets with bleeding on probing, or mucosal recession of ≥0.5 mm after surgical reconstructive treatment [51].

According to Roccuzzo et al. (2021), approximately 54% of the patients undergoing reconstructive treatments experienced disease recurrence. Significantly, the study included both compliant and non-compliant patients with SPIT, showing that peri-implant complications and implant failures were more frequent in non-compliant individuals [52].

In a study conducted by Carcuac et al., 100 patients with advanced peri-implantitis were treated surgically [53]. The goal was pocket elimination, with three test groups receiving additional therapies: systemic antibiotics, antiseptic implant surface decontamination, or both. The study evaluated outcomes at 1 and 3 years post-treatment using clinical and radiographic examinations.

Over the course of 1 to 3 years, there were notable improvements in the health of peri-implant soft tissues, including an average decrease in probing depth of 2.7 mm. Bone stability was characterized by an average bone loss of 0.04 mm. Additionally, the advantages of using systemic antibiotics were mainly observed in implants with altered surfaces, but these advantages decreased after the first year [53]. There are various types of modified titanium surfaces for dental implants, such as sandblasted surfaces, acid-etched, sandblasted large-grit acid-etched (SLA), plasma-sprayed, anodized, and hydrophilic [54]. In the study by Carcuac et al., non-modified implant surfaces presented with more favorable clinical outcomes after surgical therapy [53]. Despite their initial advantages in osseointegration, the modified surfaces appeared to be more prone to complications, such as infection or disease recurrence, particularly in cases where systemic antibiotics were not used when peri-implantitis had been established.

Lastly, 35% of all treated implants presented with probing pocket depth > 5 mm at 3 years [53]. In another study by the same group, 44% of the implants analyzed experienced disease recurrence, characterized by bone loss exceeding 1 mm, which either led to the repetition of therapy or explantation [55].

Serino et al., in a 10-year retrospective study, found that for implants reaching the endpoints of probing pocket depth ≤ 4 mm and without bleeding on probing and/or suppuration following peri-implant surgery, 84% remained healthy throughout the observation period. However, 29% of the implants treated showed disease progression. Additionally, there were a total of 11 implant failures observed throughout the entire monitoring period [56].

The clinical management of implants with advanced peri-implantitis lesions (50% or more of the total length of the implant) is recommended to be explantation [57].

The findings from these various studies highlight that peri-implantitis treatment does not always result in long-term resolution, and the disease frequently persists or recurs, often leading to significant complications. Despite efforts to manage the condition, recurrence rates remain substantial. Furthermore, the economic and clinical burden associated with peri-implantitis is important, as recurrence frequently necessitates additional treatments, implant removal, or replacement, increasing costs and complicating patient care.

Modern bone reconstruction techniques, such as guided bone regeneration (GBR) and ridge augmentation, offer predictable and successful outcomes in preserving and regenerating alveolar bone [22]. Moreover, graftless options, such as the use of short implants, present similar results of success and survival in recent studies. A systematic review and meta-analysis of randomized controlled clinical trials (RCTs) compared the success of rehabilitation of the atrophic posterior maxilla with either short or long implants. Twenty-two RCTs were assessed with 667 patients and 1595 implants (short implant: 767, long implant: 835). No significant difference in implant survival rate was recorded for short and long implants (at patient level: RR: 1.01, 95% CI = 0.52–2.0, *p* = 0.87, I^2^ = 0%, at implant level RR = 1.09, 95% CI = 0.6–2.0, *p* = 0.7, I^2^ = 0%). Similarly, marginal bone resorption was reported with no difference for short and long implants (MD = 0.16, 95% CI = −0.23 to −0.08, *p* = 0.00, I2 = 74.83%). Biological complications were marginally higher for long implants (RR = 0.48, 95% CI = 0.23–0.8, *p* = 0.13, I^2^ = 29.11%). and prosthetic complications were marginally higher for short implants (RR = 1.56, 95% CI = 0.85–3.15, *p* = 0.43, I^2^ = 0%). The authors concluded that short implants are a suitable alternative to long implants with sinus grafts for the rehabilitation of the posterior atrophic maxilla.

Therefore, the argument for preventive tooth extraction to avoid further bone loss is unjustified. With the proper application of these techniques, clinicians can manage bone defects effectively, eliminating the need for premature extractions and maintaining the tooth’s function for the long term. Consequently, extraction is not the only solution when bone loss is present.

## 4. Summary of Findings

The dilemma of whether to preserve a periodontally compromised tooth or opt for extraction and dental implant placement is a complex decision faced by clinicians daily. Dental implants have become a standard of care for failing teeth due to their high survival rates, yet complications such as peri-implantitis are common, affecting up to 22% of implants within just three years. The mechanical approach often overshadows the biological considerations, especially in patients with periodontal disease.

Periodontal therapy aims to preserve teeth, and studies show that even teeth with furcation involvement can still be saved with proper care, achieving survival rates of over 90% within 5–9 years. For example, Nibali et al. demonstrated that molars with furcation involvement achieved survival rates of 94–100% after 4–7.5 years with regenerative techniques. However, the severity of the furcation involvement on a horizontal level is proportional to an increase in the loss rate, reaching 30% for molars with degree III. No matter the therapeutic approach, supportive periodontal therapy is critical in the long-term preservation of compromised teeth.

Implant survival rates may be 90.5–100% at five years and 85.5–100% at 10 years, but survival alone is no longer considered the primary measure of success. Peri-implant complications, especially peri-implantitis, are highly prevalent, with recurrence rates as high as 44–54% even after regenerative treatment. Smoking and history of periodontal disease are the most important factors for disease onset.

The argument for preventive tooth extraction to avoid further bone loss is not justified, as modern bone reconstruction techniques offer highly predictable and successful alternatives. Procedures like guided bone regeneration and ridge augmentation have demonstrated the ability to preserve and regenerate bone around natural teeth or in cases of inadequate bone for implant placement. These regenerative techniques reduce the need for extraction and maintain the function of natural teeth, making preventive tooth extraction an overly aggressive approach in many cases.

Moreover, such interventions allow for the restoration of bone structure, addressing existing bone loss, and supporting long-term dental health. With proper care and advanced methods in bone regeneration, keeping natural teeth can be a viable option even in situations previously considered for extraction.

Furthermore, economic factors and patient preferences should also be taken into account. While implant placement can also be costly, surgical and non-surgical periodontal treatments may offer a less invasive and potentially more cost-effective solution, depending on the situation. Patients might favor retaining their natural teeth, provided they understand the long-term benefits and success rates of periodontal treatments, rather than choosing extraction prematurely.

## 5. Conclusions

While dental implants are reliable with high survival rates, complications such as peri-implantitis can challenge long-term success, requiring retreatment or explantation, occurring at a very high prevalence within the first five years after installation. According to a recent meta-analysis, the prevalence of peri-implantitis on implant level ranged from 1.1% to 85.0% and the incidence from 0.4% within 3 years to 43.9% within 5 years, respectively. The median prevalence of peri-implantitis was 9.0% (adjusted for sample size [SSA] 10.9%) for regular participants of a prophylaxis program, 18.8% (SSA 8.8%) for patients without regular preventive maintenance, 11.0% (SSA 7.4%) for non-smokers, 7.0% (SSA 7.0%) among patients representing the general population, 9.6% (SSA 9.6%) for patients provided with fixed partial dentures, 14.3% (SSA 9.8%) for subjects with a history of periodontitis, 26.0% (SSA 28.8%) for patients with implant function time ≥5 years, and 21.2% (SSA 38.4%) for ≥10 years. On a medium and medium-high level of evidence, smoking (effect summary Odds Ratio [OR] 1.7, 95% CI 1.25–2.3), diabetes mellitus (effect summary OR 2.5; 95% CI 1.4–4.5), lack of prophylaxis, and history or presence of periodontitis were identified as risk factors of peri-implantitis. There is medium-high evidence that the patient’s age (effect summary OR 1.0, 95% CI 0.87–1.16), gender, and maxillary implants are not related to peri-implantitis. Periodontal therapy, comprising all steps (1,2,3) and supportive therapy, offers a viable, cost-effective alternative to implants for teeth with furcation involvement or severe attachment loss, especially when regular follow-up and optimal oral hygiene is ensured. Tooth survival of severely compromised teeth reaches a 10-year follow-up at very high percentages, and satisfactory numbers are also noted for longer follow-up periods [58] The decision between tooth preservation and implant placement should thus be based on a holistic approach, considering not just the ability for implant placement but also the patient’s periodontal health and local and systemic risk factors for implant failure. Primordial prevention is the first step for implant success, as stated by the new treatment guidelines on peri-implant diseases and strongly associated factors for peri-implant disease and failure, as discussed, need to be taken into great consideration prior to treatment plan execution.

## Data Availability

No new data were created or analyzed in this study.
